# Bayesian Regularized Artificial Neural Network Model to Predict Strength Characteristics of Fly-Ash and Bottom-Ash Based Geopolymer Concrete

**DOI:** 10.3390/ma14071729

**Published:** 2021-04-01

**Authors:** Sakshi Aneja, Ashutosh Sharma, Rishi Gupta, Doo-Yeol Yoo

**Affiliations:** 1 Department of Civil Engineering, University of Victoria, Victoria, BC V8W 2Y2, Canada; r.sharmaashutosh@gmail.com (A.S.); guptar@uvic.ca (R.G.); 2 Department of Architectural Engineering, Hanyang University, Seoul 04763, Korea; dyyoo@hanyang.ac.kr

**Keywords:** geopolymer concrete, fly-ash, bottom-ash, neural network, sustainability, industrial waste management

## Abstract

Geopolymer concrete (GPC) offers a potential solution for sustainable construction by utilizing waste materials. However, the production and testing procedures for GPC are quite cumbersome and expensive, which can slow down the development of mix design and the implementation of GPC. The basic characteristics of GPC depend on numerous factors such as type of precursor material, type of alkali activators and their concentration, and liquid to solid (precursor material) ratio. To optimize time and cost, Artificial Neural Network (ANN) can be a lucrative technique for exploring and predicting GPC characteristics. In this study, the compressive strength of fly-ash based GPC with bottom ash as a replacement of fine aggregates, as well as fly ash, is predicted using a machine learning-based ANN model. The data inputs are taken from the literature as well as in-house lab scale testing of GPC. The specifications of GPC specimens act as input features of the ANN model to predict compressive strength as the output, while minimizing error. Fourteen ANN models are designed which differ in backpropagation training algorithm, number of hidden layers, and neurons in each layer. The performance analysis and comparison of these models in terms of mean squared error (MSE) and coefficient of correlation (R) resulted in a Bayesian regularized ANN (BRANN) model for effective prediction of compressive strength of fly-ash and bottom-ash based geopolymer concrete.

## 1. Introduction

With a focus on decarbonization, different ways of reducing greenhouse gas emissions are being constantly explored [1]. The construction industry typically requires a huge amount of energy for its products and services and, therefore, is tagged as a carbon-intensive sector. Hence, it significantly challenges sustainable growth. In the entire spectrum of the construction industry, the production of cement alone produces the largest amount of carbon dioxide and is the second largest source of CO_2_ emission worldwide. In this regard, geopolymer concrete offers a potential solution to completely overtake the role of cement in the construction industry.

The term ‘geopolymer’ was first used in Davidovits’ work relating to the formation of polymeric Si-O-Al bonds from a chemical reaction of alkali silicates with aluminosilicate precursors. As per Duxson’s model [1], the process of geo-polymerization involves three steps: (1) the dissolution of aluminosilicate materials and the release of silicate and aluminate monomers (Si(OH)_4_)- and (Al(OH)_4_); (2) initial gels (mono cross-linked systems) produced by co-sharing of oxygen atoms from the reactive silicate and aluminate monomers, a process known as condensation; (3) the initial gels are converted into geopolymer gels in the last stage, a process known as polycondensation. Just like ordinary concrete, geopolymer concrete can be developed by adding aggregates, and prepared with waste materials such as fly ash, glass granulated blast slag, rice husk ash to form geopolymers. By using different industrial waste materials, two problems, viz, (1) high demand for cement, (2) industrial waste management, can easily be solved.

There is a major difference between the hydration process of cement and the polymerization process in geopolymers. This is primarily due to the usage of different precursor materials. Different industrial waste materials such as glass granulated blast furnace slag (GGBFS) [2,3,4,5,6,7,8], Fly Ash (FA) [9,10,11,12,13], and metakaolin (MT) [14,15,16,17,18,19,20,21,22,23] have been used as source materials for developing geopolymer concrete, as reported by the researcher community. Geopolymer concretes are usually less workable, so much so that a nominal 90 mm slump is considered as necessary [24]. With the addition of slag in geopolymer concrete, the workability of the mix gets reduced [25]. Further, it accelerates the geo-polymerization, significantly reducing the initial and final setting times. This could be due to the formation of additional C-S-H gel during geo-polymerization [3]. The mechanical properties of GGBFS based geopolymer concrete cured under ambient conditions is greater than that of normal concrete [6]. Metakaolin based geopolymer greatly accelerates the geo-polymerization due to its high reactivity and, hence, reduced initial and final setting times are achieved [14,17]. The fine particle size of metakaolin fills pores in the matrix and significantly reduces porosity, resulting in a densified microstructure [17,18,19]. On the other hand, surface cracks are developed at elevated temperatures due to the movement of water from the matrix to the surface, resulting in increased water absorption [23]. The characteristics of FA based geopolymer primarily depend on the purity of raw materials and the concentration of alkali solutions, the physiochemical properties of fly ash, alkali activators, and curing conditions [10,13]. Different gels can be formed by varying the Si/Al ratio and alkali solutions, influencing the final geopolymer structure and controlling the ionic transport. Further, the hydrolyzation of fly ash depends on the alkali solution and hence the porosity of the geopolymer structure. This further impacts the movement of moisture and alkali from the geopolymer into ion solution, enhancing its mechanical strength and durability. FA based geopolymer exhibits promising resistance to chloride, sulphate and acid solutions [12]. It exhibits good efflorescence and freeze-thaw resistance as well [11].

The mechanical characteristics of any geopolymer concrete depend on multiple variables such as precursor ingredients, type of silicates, concentration, type of material as cement replacement and its quantity, amount of superplasticizers, type of curing conditions, time of curing, etc. Multi-variability of inputs complicates the process of optimization in determining proper mixture proportion while synthesizing geopolymers. Therefore, the expected results can only be obtained by properly choosing the combination of materials and correctly selecting the mix proportions. This is normally a cumbersome process involving large-scale laboratory-based experiments, a large number of materials, time, labour, and high cost [26]. This is reflected in some of the published works [27,28,29,30] wherein numerous mixes were made to find a suitable proportion for GPC of the desired characteristics, while others developed a multi-step methodology [31] to achieve high 28-day compressive strength.

Compressive strength is one of the main design parameters as mentioned in design codes and standards that indicates the ability of concrete to withstand loads. Hence, numerous empirical relationships have been reported and published for predicting the compressive strength of different types of geopolymer concrete. Traditional statistical models are ineffective in considering the actual scenarios of concrete with different constituents and the results cease to be accurate when new data differing from the original data is used. This is primarily because conventional statistical models are built with fixed equations based on limited inputs. Recently, artificial neural networks (ANN) have gained popularity in various civil engineering problems such as drying shrinkage, concrete durability, and workability of different concretes [32,33,34,35,36,37]. The ability to draw relevant inferences makes ANN a very effective prediction method. Many researchers have used ANN to predict the compressive strength of different types of geopolymer concrete with significant success [32,38,39,40]. However, the use of ANN for GPC and the influence of bottom ash (BA) as a replacement for cement and sand in fly ash-based geopolymer concrete has rarely been reported.

This study aims to investigate the influence of industrial waste materials such as BA on the characteristics of alkali-activated geopolymer concrete. The numerical prediction modelling for compressive strength of geopolymer concrete has been implemented with ANN. For that purpose, three algorithms, namely Levenberg Marquardt backpropagation, Bayesian Regularisation backpropagation and Scaled conjugate gradient backpropagation, have been utilized. The efficacy of each algorithm for prediction analysis has also been evaluated.

## 2. Artificial Neural Network Architecture

**Figure 1:**
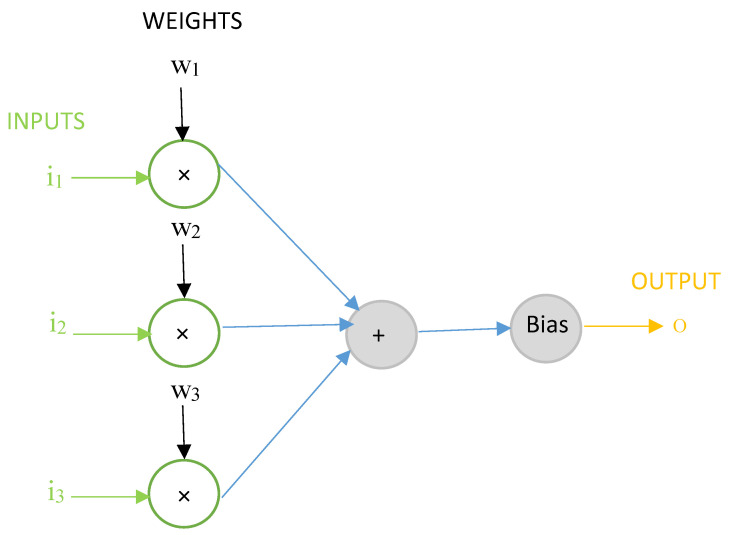
A computational neuron in the artificial neural network (ANN) model.

ANN is a machine learning prediction model that can predict the expected output when trained with a data-set of inputs and output. An artificial neuron is the computational unit in ANN and therefore is also known as a “computational neuron”. A schematic of a computational neuron in an ANN model with three inputs and one output is shown in Figure 1. The basic operation in an ANN model involves the multiplication of input features *i*_1_, *i*_2_, …, *i_n_* with weights *w*_1_, *w*_2_, …, *w_n_* to calculate a sum of weighted inputs *i*_1_ *w*_1_ + *i*_2_ *w*_2_ + … + *i_n_x_n_*. The sum of weighted inputs is compared with a certain threshold value also known as ‘bias’ and an output *o* is generated. If the weighted sum of inputs is greater than or equal to the bias, an output signal is transmitted further in the network, otherwise not.

**Figure 2:**
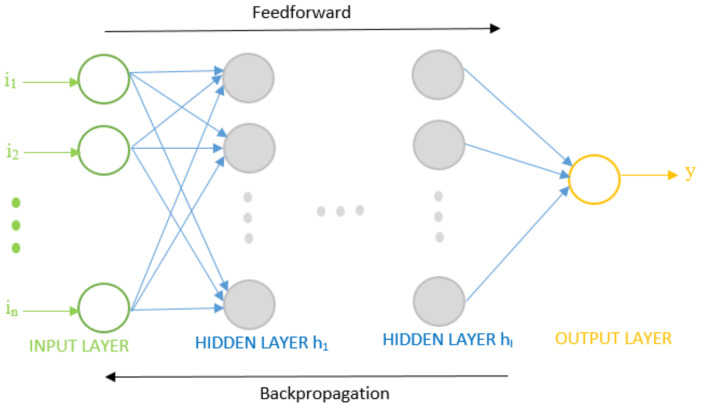
System model of multilayer feedforward neural network.

A feedforward network is an ANN with a forward flow of information from an input layer to an output layer through one or more computational layers known as hidden layers. Figure 2 shows the system model of a multilayer feedforward neural network. The input layer comprises nodes that represent the input parameters or features in the data fed into a network model. The hidden layer has numerous neurons that operate on the weighted inputs using an activation function. The output layer comprises one or more output nodes that utilize an activation function to give the estimated output *y*. The neurons in consecutive layers are connected. The term multilayer signifies the number of layers with an activation function. A feedforward network is said to be a single-layer network if its input layer is directly connected to the output layer. A feedforward network is said to be a two- or three-layer network if its input layer is connected to the output layer through one or two hidden layers, respectively. The input features in data are represented by *i*_1_, *i*_2_, …, *i_n_* where *n* is the number of input features. Hidden layers are represented by *h*_1_, *h*_2_, …, *h_l_* where *l* is the number of hidden layers. Each hidden layer can have multiple neurons as their elementary unit, as represented by shaded circles. The output layer is represented by a single functional unit that estimates the actual output or target *t* in experimental data.

A backpropagation algorithm can be effectively used to train feedforward neural networks to predict an expected output that closely matches the target. The network training using backpropagation is an iterative process where each forward flow of information is followed by a backward pass that adjusts weights and biases. In each forward pass of information, the cost function which is a function of error between output and target is calculated. Gradients are obtained by differentiating the cost function for independent weights. In each iteration, gradients are calculated as a result of a chain rule and adaptive weights and biases are fed to the network to be used by the next forward flow of information. The backpropagation algorithm is aimed at reducing this cost function by finding a local minimum. This process is continued until error is minimized for efficient training and hence a better prediction model [41].

In this study, a multilayer feedforward neural network is designed and trained with Levenberg-Marquardt (LM), Bayesian Regularization (BR), and scaled conjugate gradient (SCG) backpropagation algorithms separately to identify an efficient model that can predict the compressive strength of geopolymer concrete. The multilayer feedforward neural network in this research uses the sigmoid activation function in the hidden layers and linear activation function in the output layer. Network training with LM and BR backpropagation algorithms involves Jacobian calculations while training with SCG backpropagation algorithm involves gradient calculations. LM backpropagation is the least time-consuming algorithm for training moderate-sized neural networks but consumes maximum memory. Training is stopped when the network’s performance is not improving, or the network is not generalizing well. BR backpropagation consumes most of the time but can be applied to small or noisy datasets. Training is continued to the point when optimum weights are found. SCG backpropagation consumes the least memory and can be applied to any network. Training is stopped when the network’s generalization is not improving further. 

The BR backpropagation algorithm avoids both overfitting and overtraining as the network trains on effective network parameters or weights and does not consider the irrelevant parameters. Equation (1) provides the training objective function F(ω) used by BR, where S_ω_ is the sum of squared network weights and S_e_ is the sum of network errors. A combination of squared errors and weights is minimized until the optimum combination is achieved for which the network generalizes well. At that point, training is stopped [42].

F(ω) = αS_ω_ + βS_e_ (1)

The input and output parameters of geopolymer concrete specimens used to design a multilayer feedforward neural network-based prediction model in Matrix Laboratory (MATLAB) are given in Table 1. The specifications of geopolymer concrete represented by *i*_1_, *i*_2_, …, *i*_11_ are considered as eleven input features in the input layer of the network. Even though the features are quite commonly used in the context of geopolymer concrete, further details about what these features mean is reported in the literature [43,44]. This is not described here in order to maintain brevity. The output layer comprises a single neuron that predicts the expected compressive strength *y*, known as output, that is mapped to the actual compressive strength of geopolymer concrete known as target *t*. Table 1 also shows the minimum and maximum values of input and output data considered in this study.

## 3. Data Preparation

To develop an effective ANN model, the data was collected as input parameters as well as output parameters from previous research works published on fly ash and bottom ash-based geopolymer [9,45,46,47,48,49,50,51,52,53,54,55,56,57]. Further, some mixes were also developed in the laboratory to collect data. It is to be noted that compressive strengths of 7 days, 14 days, and 28 days were considered for developing the ANN model.

### 3.1. Data from Literature Sources

A total of 46 sets of experimental data from 15 research papers (details provided below) was collected as input to develop the ANN strength model. For this work, a total of 11 input parameters were included. These include coarse aggregates (CA), fine aggregates (FA), fly ash (FAH), bottom ash (BAH), sodium silicates (SS), sodium hydroxides (SH), sodium silicate and sodium hydroxide ratio (SS/SH), precursor powder and liquid ratio (L/S), curing time (CT) and amount of superplasticizer (S). The basic premise behind selecting these parameters is due to to their direct influence on the matrix, and consequently mechanical properties, of GPC (mainly its compressive strength). Table 2 gives the sources of data used in the study.

### 3.2. Data from Experiments

From the literature review, it can be seen that not many data sources are available for modelling the FA and BA based GPC. Furthermore, it can be seen that there is a wide variation in the values reported in the literature. Using such information for further mix optimization would not be straightforward. Considering experimental data as the key for validation of numerical models, extensive experimentation was carried out in the laboratory. The experiments involved three different kinds of mixes: (1) fly ash-based geopolymer mix; (2) fly ash-based geopolymer mix with bottom ash fine aggregates; (3) fly ash based geopolymer with bottom ash as a replacement for fly ash itself.

For the production of GPC, class F fly ash as a pozzolanic material obtained from Bathinda coal power plant in India was used. The physical properties of fly ash were: specific gravity: 2.4, bulk density (kg/m^3^): 700, surface area (kg/m^2^):19,000. The chemical composition of fly ash is given in Table 3. Bottom-ash used in this study was also obtained from the above noted thermal power plant. The specific gravity and water absorption of bottom ash was 1.39 and 31.48%. The chemical properties of bottom ash are also given in Table 3.

NaOH (SH) and Na_2_SiO_3_ (SS) were used for activation of the precursor material. Anhydrous SH powder was dissolved in water to produce SH solutions with varying molarities (10 M, 12 M, 14 M). The solution was prepared 24 h before its usage. Later, SS solutions were mixed with SH solutions at different mass ratios (1.5, 2, 2,5).

Fine aggregates and coarse aggregates obtained from local sources in Jalandhar (Punjab, India) had a relative dry density of 2.671 and 2.713, respectively, and a water absorption ratio of 0.79% and 0.69%, respectively. The coarse aggregates with a maximum size of 12.5 mm were used for preparing GPC and ordinary Portland cement concrete (OPC). The fine aggregates used in both OPC and GPC were medium-coarse sand which was suitable for multipurpose use including concrete mixtures.

The solid constituents of GPC, i.e., the aggregates, fly-ash, and bottom-ash, were first mixed in the dry condition in a rotary drum mixer for about 1 min. Next, the alkali solution was added to the solids and mixed for about 3 min, followed by 3 min rest period, then followed by 2 min of final mixing. The mixture was placed into moulds of size 150 × 150 × 150 mm and vibrated using a table vibrator for 30 s to discharge air bubbles to the surface. Then, the moulds were covered with a plastic sheet in a lab environment (approximate relative humidity range of 45–70% and approximate temperature range of 5 °C to 15 °C) and demoulded after 24 h A total of 55 mixes were prepared and the compressive strength after 7, 14, and 28 days was evaluated. The various mix designs and the experimentally evaluated compressive strength values are given below in Table 4. It should be noted that other experimental results will be reported by authors in upcoming manuscripts. The experimental results indicate that the increased concentration of sodium hydroxide (SH) from 12 to 16 improved the compressive strength for all the mix design samples [58,59]. Similarly, the increasing ratio of the sodium silicate to sodium hydroxide exhibited increased compressive strength of specimens of all mix designs. It should be noted that the ratio of alkaline to that of fly-ash was fixed at 0.4 for the entire study as it exhibited the best results in the pilot studies [60]. From the mix design, it can be seen that bottom ash was replaced with cement for GPC-1, GPC-2, and GPC-3 by 0%, 20%, and 40%. The results indicate that GPC-1 with precursor as fly ash alone exhibits the highest compressive strength which further increases with SS/SH ratio and the SH concentration. The replacement of fly-ash as a precursor with 20% and 40% reduced the compressive strength by 25% and 35% [60]. This reduction in compressive strength may be attributed to lesser polymerization of bottom ash particles in comparison to fly ash particles [61,62]. However, the replacement of fine aggregates with bottom ash for mix designs GPC-4, GPC-5, and GPC-6 by 20%, 40%, and 50% exhibited better compressive strength values than before. All the samples with 20% replacement of bottom ash with fine aggregates exhibited higher compressive strength values than the GPC-1. The results from GPC-4 samples with SH concentration of 16 exhibited 23% higher values of compressive strength than the GPC-1. All other samples from GPC-5 and GPC-6 exhibited far lesser values of compressive strength. This may be attributed to the large and porous structure of bottom ash particles inducing internal voids and cracking under loading.

## 4. Results and Discussions

**Figure 3:**
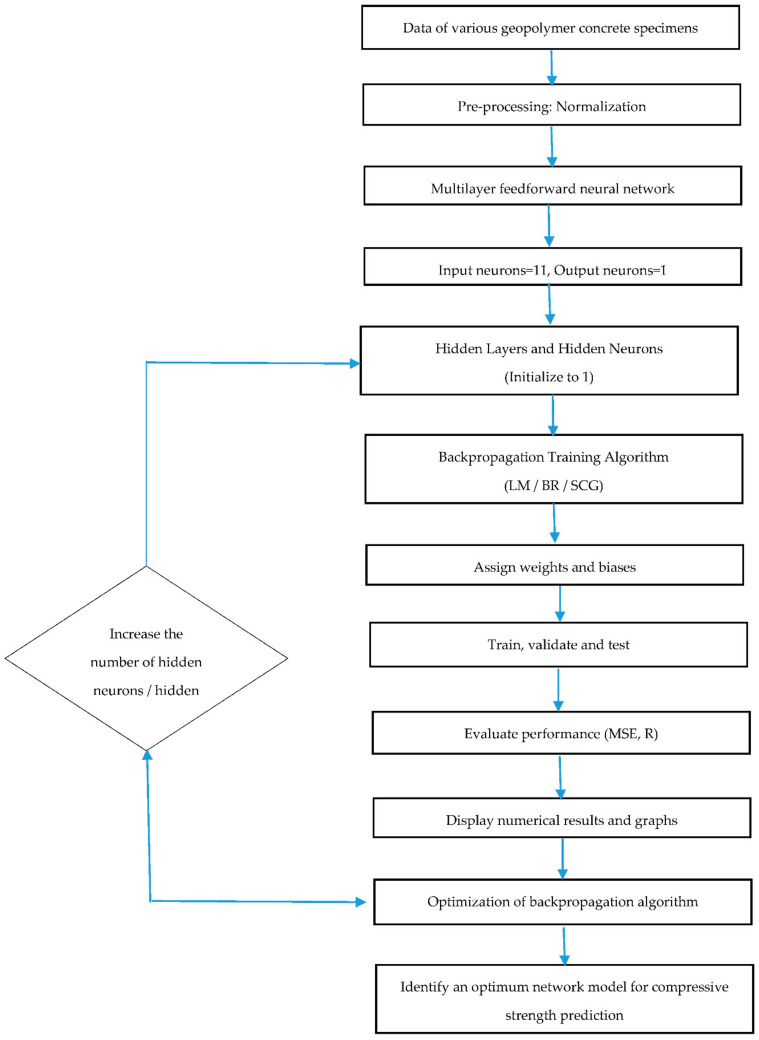
Step-by-step procedure to predict the compressive strength of geopolymer concrete.

The research methodology for identifying a suitable ANN model to predict the compressive strength of geopolymer concrete is shown in Figure 3. The data of geopolymer concrete specimens, as reported in Table 1, is normalized and sampled as 70% for training, 15% for validation, and 15% for testing. The training data is presented to the network in order to predict output compressive strength closer to target compressive strength, validation data measured network generalization to keep a check on training, and testing data measured network’s performance during and after training. The network optimization is aimed at obtaining a hypothesis function that predicts the compressive strength of geopolymer concrete with a minimum difference between output and target. This involves various trials and rigorous network training by varying the number of hidden layers between the input and output layer and neurons in each hidden layer.

The network’s performance is analysed by training with different backpropagation algorithms such as LM backpropagation, BR backpropagation, and SCG backpropagation. The model is trained by reducing mean squared error (MSE) and as a result, increasing coefficient of correlation (R). MSE is calculated by averaging the squares of the difference between output and target. An MSE of zero signifies no error i.e., perfect condition. R represents regression values and measures the relationship between output and target. A close relationship has R = 1 in a perfect scenario. An extensive search was carried out in our study to find the optimum hidden layers, hidden neurons, and backpropagation algorithm in an endeavour to build a reliable ANN model for the prediction of compressive strength. Different models of ANN are presented in the following discussion which is applied to geopolymer concrete data to determine an optimum model that predicts compressive strength with the lowest MSE and highest correlation between output and target.

### 4.1. Prediction Evaluation of Compressive Strength

**Figure 4:**
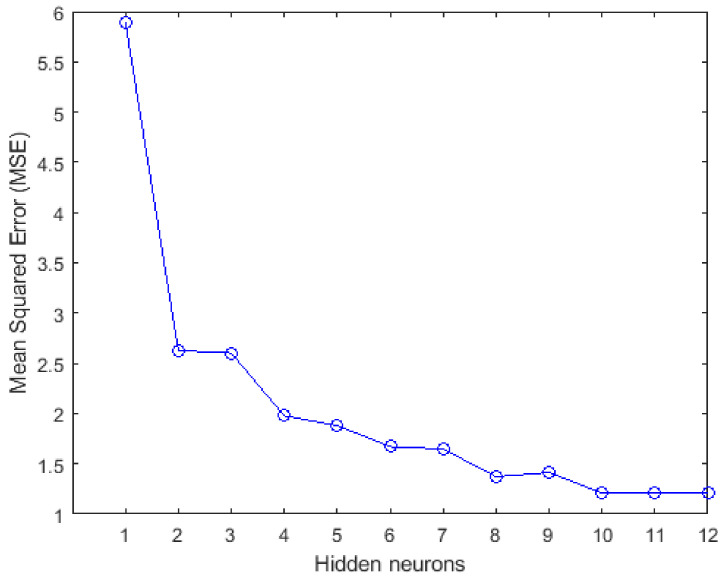
Effect of increasing hidden neurons on the network’s performance to predict the compressive strength.

Firstly, a two-layer feedforward neural network comprising an input layer, a hidden layer, and an output layer and trained with BR backpropagation algorithm is programmed. The network’s performance is analysed by checking its ability to predict the compressive strength of geopolymer concrete with low MSE. Figure 4 shows the effect of hidden neurons on the performance of the BR trained network. It was observed that MSE decreases with an increase in the number of hidden neurons till 10, after which there is no significant decrease. This implies that the estimated compressive strength predicted by the network model can be improved by optimizing the hidden neurons. For instance, the compressive strength of geopolymer concrete can be predicted with an MSE of 1.8809 considering 5 hidden neurons, and with an MSE of 1.2098 considering 10 hidden neurons.

To achieve a better prediction of compressive strength, various ANN models are designed in this study by considering different backpropagation algorithms and varying the number of hidden layers and hidden neurons. Table 5 enlists ANN-I to ANN-XIV models based on two, three- and four-layer feedforward neural networks trained with BR, LM, and SCG backpropagation algorithms. ANN-I to ANN-VI are two-layer feedforward network models with a single hidden layer between the input and output layer. ANN-VII to ANN-X are three-layer feedforward network models with two hidden layers between the input and output layer. ANN-XI to ANN-XIV are four-layer feedforward network models with three hidden layers between the input and output layer.

**Figure 5:**
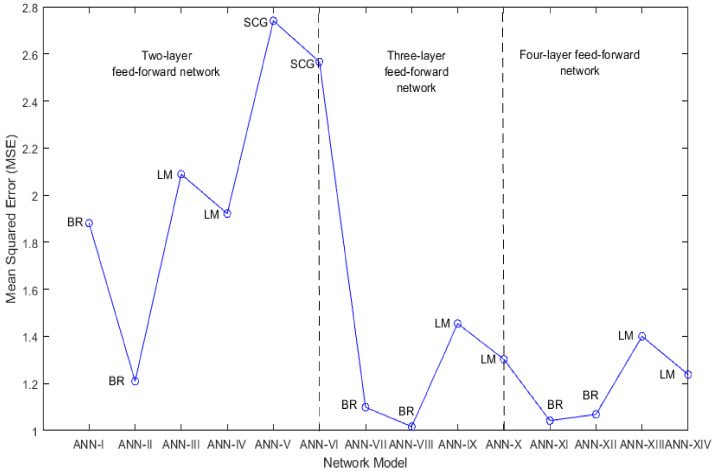
Mean squared error (MSE) performance comparison of two, three- and four-layer feedforward network models.

**Figure 6:**
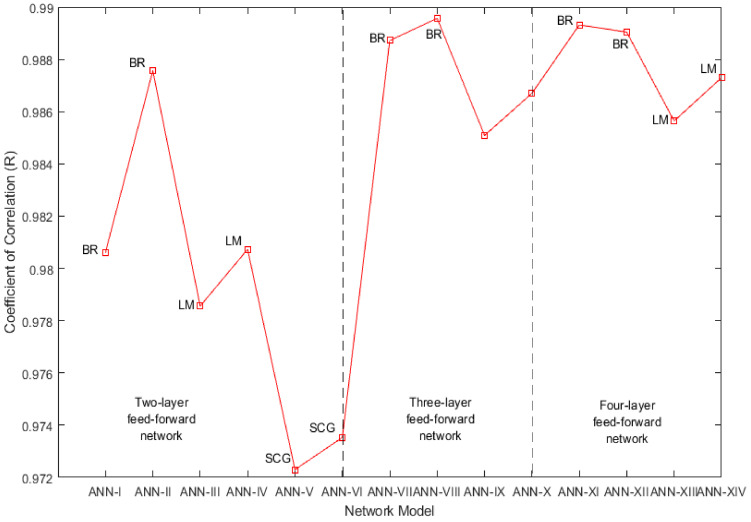
Correlation coefficient comparison of two, three- and four-layer feedforward network models.

The compressive strength prediction ability of the ANN-I to ANN-XIV network models is evaluated by analysing both MSE and R. Figure 5 and Figure 6 show the MSE and R performance of these network models. Considering two-layer feedforward network models ANN-I to ANN-VI, it is observed that MSE_BR_ < MSE_LM_ < MSE_SCG_ and R_BR_ > R_LM_ > R_SCG_. This implies that network models trained with BR backpropagation are effective in predicting compressive strength. The network models trained with SCG backpropagation failed to provide a good prediction. Considering three-layer feedforward network models ANN-VII to ANN-X, it is observed that MSE decreases significantly on increasing the hidden layers, resulting in a better prediction. Further, the network models trained with BR and LM algorithms show a similar coefficient of correlation i.e., R_BR_ ≈ R_LM_, but BR trained networks have a better prediction of compressive strength than LM trained networks by reducing MSE, i.e., MSE_BR_ < MSE_LM_. Considering four-layer feedforward network models ANN-XI to ANN-XIV, a slight reduction in MSE of LM trained networks is observed due to the addition of another hidden layer but there is no improvement in MSE of BR trained networks indicating no need for increasing hidden layers beyond 3. Again, BR trained networks outperform LM trained networks by predicting compressive strength with lesser MSE, i.e., MSE_BR_ < MSE_LM,_ and a comparable coefficient of correlation, i.e., R_BR_ ≈ R_LM_.

During network training, LM and SCG backpropagation algorithms experience repetitive validation failures and training is stopped after six validation failures. On the contrary, a network trained with BR backpropagation can perform well on the validation data set, indicating the flexibility of prediction for unknown data. A comparison of all network models establishes the conclusion that the ANN-VIII model which is a three-layer Bayesian regularized artificial neural network (BRANN) with 10 neurons in each hidden layer is effective in predicting compressive strength of geopolymer concrete with the least MSE (1.017) and the highest R of 0.99. However, it should be noted that this performance exhibited by BRANN is at the cost of more epochs (a measure of the number of times the algorithm uses training vectors to give a hypothesis for prediction), which should not be a problem in the current era of robust and efficient hardware. This implies that the BR backpropagation algorithm improves the training of feedforward neural networks when the number of hidden layers and neurons in each hidden layer is optimized.

### 4.2. Performance Analysis of BRANN Prediction

The performance of three-layer BRANN to predict the compressive strength of geopolymer concrete in the ANN-VIII model is verified by checking the balance between training and non-training testing patterns. It should be noted here that non-training validation data is not recommended for performance analysis as it gives a biased estimate of prediction by stopping the network’s training when performance starts to deteriorate. The intention is to get an unbiased estimate of the prediction ability of a network model built for training data by applying the same model to testing data of geopolymer concrete specimens. A network model that can predict well on testing data can predict the unknown compressive strength for any other input specifications of geopolymer concrete. However, the reliability of predicted compressive strength is dependent on the type of experimental data used for network training.

**Figure 7:**
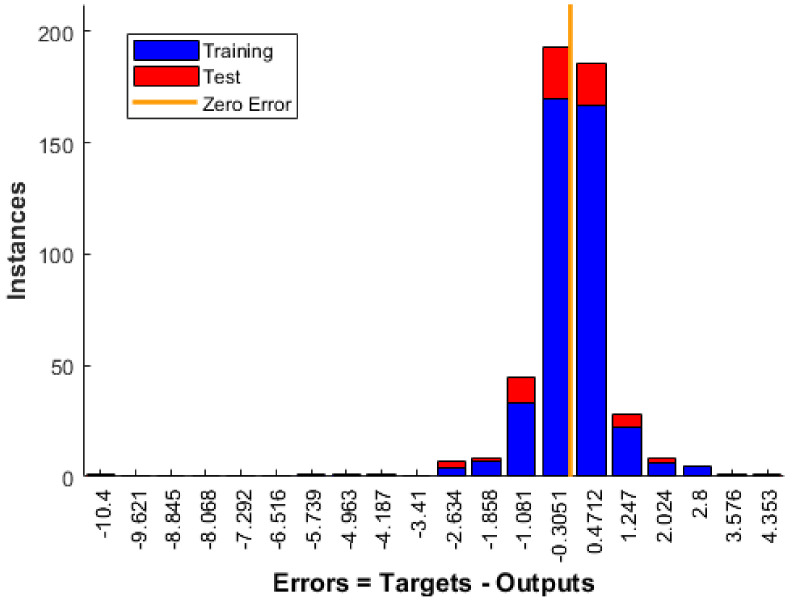
Error Histogram of three-layer Bayesian regularized ANN (BRANN) for GPC compressive strength prediction.

Figure 7 shows the error histogram obtained after training and testing the network model. The error on the x-axis specifies how predicted compressive strength (output) differs from the actual compressive strength of geopolymer concrete (target). Instances on the y-axis specify the number of geopolymer concrete specimens in the training or testing dataset with a specific error. Most of the errors after training and testing with three-layer BRANN lie in the range of −1.081 to 1.247. Further, three-layer BRANN can predict compressive strength for the majority of geopolymer concrete specimens with an error between −0.3051 to 0.4712, which is closer to the zero error line.

**Figure 8:**
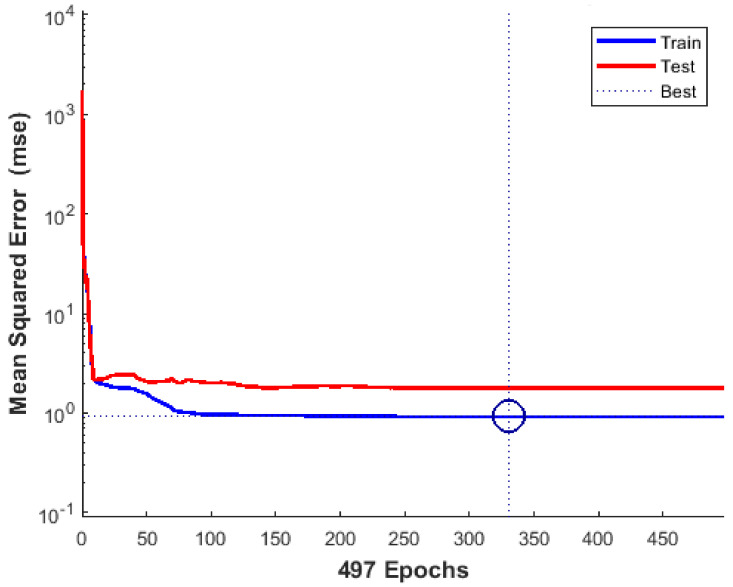
Mean squared error performance of three-layer BRANN for GPC compressive strength prediction.

Figure 8 shows the pattern of the MSE performance of three-layer BRANN for epochs during the training and testing phase. The results indicate that, as the epochs are increased, BRANN can predict GPC compressive strength with a very low MSE due to efficient training. The prediction ability of BRANN improves to 250 epochs and remains constant afterward. The best training performance in terms of lowest MSE is highlighted with a circle corresponding to prediction with MSE of 0.92263 at epoch 330. BRANN is also able to predict the compressive strength on the testing dataset with a comparable MSE, verifying the effectiveness of GPC in the network model.

**Figure 9:**
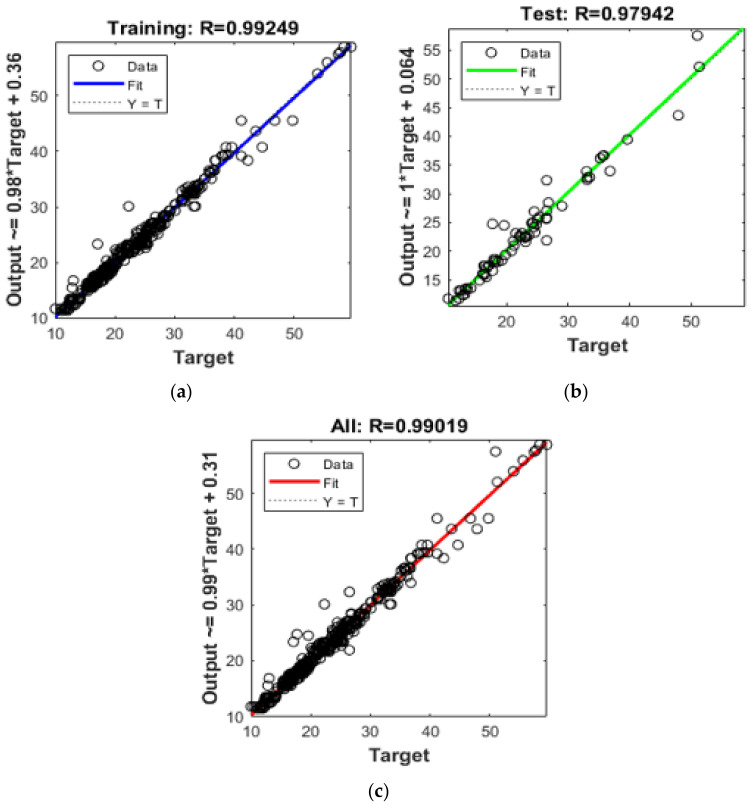
Correlation between predicted and actual GPC compressive strength for three-layer BRANN. (**a**) Training Data; (**b**) Testing Data; (**c**) All Data.

Figure 9 displays the correlation curves obtained after applying three-layer BRANN on training, testing, and complete data of GPC specimens. A perfect fitting in an ideal scenario is represented by a dashed line at an angle of 45 degrees where output compressive strength matches the target compressive strength, i.e., coefficient of correlation R = 1. The blue, green, and red lines represent the fitting for training, testing, and entire data of GPC specimens respectively. A close relationship between output and target compressive strength with the coefficient of correlation R = 0.992, 0.979, and 0.99 for training, testing, and entire data respectively is obtained, which indicates good data fitting. This indicates the efficacy of three-layer BRANN in predicting compressive strength for any other specifications of geopolymer concrete. BRANN acts as a black-box that generates output compressive strength from input GPC specifications without defining the relationship.

The trained BRANN model was further used with various input parameters. The eleven input parameters were set to minima, maxima, and median values of their respective ranges. The combination of input parameters resulted in more than seven hundred possible mixes. Based on the data produced, the mix predicting the maximum compressive strength was found as given in Table 6. High molarity of SH (16) leads to high compressive strength. This is a well-established relationship. However, it should be noted that high compressive strength can also be obtained by incorporating bottom ash. This indicates the efficacy of the produced BRANN model as optimized mixes can be identified by considering the interdependence of all 11 input features. The various mix designs with their predicted compressive strength lay the perfect foundation for experimental work.

## 5. Conclusions

In this work, different models of multilayer feedforward neural network trained with Levenberg-Marquardt, Bayesian Regularization, and Scaled Conjugate Gradient backpropagation algorithms are used for predicting the compressive strength of geopolymer concrete with fly-ash and bottom-ash. These models are trained by optimizing mean squared error and coefficient of correlation. The proposed BRANN model is based on the experimental data collected from the literature and laboratory experiments. From the study, the following conclusions can be drawn:

1. Artificial neural network-based machine learning models are capable of predicting the strength characteristics of geopolymer concrete with fly-ash and bottom-ash.
2. MSE decreases as the number of neurons in the hidden layer increases in the feedforward neural network for estimation of compressive strength. MSE can be further reduced by increasing the number of hidden layers between the input and output layers.
3. The performance analysis for a two-layer feedforward neural network shows that MSE_BR_ < MSE_LM_ < MSE_SCG_ and R_BR_ > R_LM_ > R_SCG_. In this case, it should be noted that BR backpropagation outperforms LM and SCG backpropagation algorithms for the prediction of GPC compressive strength.
4. The performance analysis for a three-layer feedforward neural network indicates that both BR and LM backpropagation algorithms show a similar coefficient of correlation, i.e., R_BR_ ≈ R_LM_, but the BR algorithm shows better performance than the LM algorithm by reducing MSE i.e., MSE_BR_ < MSE_LM_, leading to better prediction of GPC compressive strength.
5. The performance analysis for a four-layer feedforward neural network implies a slight reduction in MSE of LM trained networks due to the addition of another hidden layer, but there is no improvement in MSE of BR trained networks. Again, BR trained networks outperform LM trained networks by predicting compressive strength with lesser MSE and greater coefficient of correlation i.e., MSE_BR_ < MSE_LM_ and R_BR_ > R_LM_.
6. The study suggests that the three-layer BRANN model with 10 neurons in each layer is the suitable model for predicting GPC compressive strength with MSE of 1.017 and R = 0.99.

This work is limited to the investigation and analysis of artificial neural networks trained with backpropagation algorithms for the prediction of GPC compressive strength. The effect of temperature during curing is not considered while training the ANN model. Therefore, a possible direction for future research could be the investigation of other machine learning techniques followed by a comparative analysis of prediction performance. Furthermore, the effect of temperature on GPC compressive strength can be studied in conjunction with other input parameters.

## Figures and Tables

**Table 1:** Input and output parameters used for prediction of geopolymer concrete (GPC) compressive strength.

Variables	Representation	Range
Super plasticizer (wt.%)	*i*_1_	0–1.5
Alkaline activator/fly-ash ratio	*i*_2_	0.4–0.6
NaOH concentration (M)	*i*_3_	10–16
Na_2_SiO_3_/NaOH	*i*_4_	1–3
NaOH (wt. in gms)	*i*_5_	41–127
Na_2_SiO_3_ (wt. in gms)	*i*_6_	93–241
Fly Ash (wt. in gms)	*i*_7_	0–450
Bottom Ash (wt. in gms)	*i*_8_	0–400
Coarse Aggregates (wt. in gms)	*i*_9_	0–1400
Fine Aggregates (wt. in gms)	*i*_10_	0–700
Curing Time (in Days)	*i*_11_	0–28
Compressive Strength (in MPa)	*t*	9–65

**Table 2:** Details of data and their sources.

No.	FAH	BAH	CA	FA	SH	SS	SS/SH Ratio	SS Concentration	L/S Ratio	S	CT	CS	Source
1	310	0	1204	649	68	102	2.5	10	0.225	6.2	0–28	43	[9]
2	404–408	0	1190–1202	640–647	41	103	2.5	14–16	0.35	6	0–28	42–45	[45]
1	417	0	927	698	92	241	2.5	15	0.4	5	0–28	43	[46]
1	400	0	1293	554	45	113	2.5	14	0.4	4	0–28	44	[47]
3	408	0	1201	647	62–68	93–103	1.5	14	0.4	4	0–28	32–48	[48]
5	408	0	1168	660	68	103	1.5	10–16	0.35	4	0–28	32–49	[49]
20	298–450	0	1100–1377	500–659	29.4–108	96–162	1.5–2.5	8–14	0.5	2–4	0–28	25.6–41	[50]
1	450	0	1150	500	108	162	1.5	12	0.6	2	0–28	35.2	[51]
2	400	0	1209–1218	651–655.9	40–45.7	100–114.3	2.5	10–14	.35	4	0–28	25.6–32.5	[52]
1	310	0	1204	649	66	108	2.5	10	0.35	4	0–28	41	[53]
1	409	0	1256	591	41	102	2.5	8	0.35	6	0–28	39	[54]
1	410	0	1100	590	40	100	2.5	14	0.55	6	0–28	38	[55]
6	414	0	1091	588	60–80	104–138	1–2	10–20	0.5	-	0–28	39–46	[56]
1	0	400	1216.1	540	66.7	133.3	2	8	0.5	8	0–28	49.3	[57]

(FAH: Fly ash, BAH: Bottom Ash, CA: Coarse aggregates, FA: Fine aggregates, SS: sodium silicate, SH: sodium hydroxide, SS/SH: Sodium Silicate and Sodium hydroxide ratio, L/S: Precursor powder and Liquid ratio, CT: Curing time, S: Superplasticizer, CS: Compressive Strength).

**Table 3:** Chemical composition of by-products (source: Bathinda coal power plant).

Compounds	SiO_2_	Al_2_O_3_	Fe_2_O_3_	CaO	MgO	SO_3_	Na_2_O	K_2_O	TiO_2_	P_2_O_5_	Mn_2_O_3_
Fly Ash (%)	58.11	27.21	5.23	2.14	0.72	NA	0.5	0.5	N/A	N/A	N/A
Bottom Ash (%)	56.44	29.24	8.44	0.75	0.40	0.10	0.09	1.29	2.89	0.2	0.14

**Table 4:** Mix proportions for Fly ash-based geopolymer concrete with bottom ash as replacement of fly ash & fine aggregates.

Mix Number	S	SS+SH/FAH	SH(M/L)	SS/SH	SH	SS	FAH	BAH	CA	FA	CS (MPa)
GPC-1 (100% FAH)	0.5	0.4	12	1.5, 2, 2.5	85.16	125.74	388	0	1170	630	35.5, 37.1, 39.9
	0.5	0.4	14	1.5, 2, 2.5	66.2	141.1	388	0	1170	630	36.2, 39.2, 41.4
	0.5	0.4	16	1.5, 2, 2.5	55.4	150.3	388	0	1170	630	36.2, 38.3, 47.1
GPC-2 (80% FAH + 20% BAH)	0.7	0.4	12	1.5, 2, 2.5	85.16	125.74	310	78	1170	630	27.7, 27.9, 29.1
	0.7	0.4	14	1.5, 2, 2.5	66.2	141.1	310	78	1170	630	31, 31.1, 31.1
	0.7	0.4	16	1.5, 2, 2.5	55.4	150.3	310	78	1170	630	33, 30, 29
GPC-3 (60% FAH + 40% BAH)	0.9	0.4	12	1.5, 2, 2.5	85.16	125.74	232	156	1170	630	23.5, 25.8, 25.2
	0.9	0.4	14	1.5, 2, 2.5	66.2	141.1	232	156	1170	630	28.7, 26.9, 25.3
	0.9	0.4	16	1.5, 2, 2.5	55.4	150.3	232	156	1170	630	28.4, 29.3, 29.6
GPC-4 (100% FAH + 20% BAH)	0.7	0.4	12	1.5, 2, 2.5	85.16	125.74	388	126	1170	504	36, 35.8, 33.2
	0.7	0.4	14	1.5, 2, 2.5	66.2	141.1	388	126	1170	504	42.3, 47.2, 49.8
	0.7	0.4	16	1.5, 2, 2.5	55.4	150.3	388	126	1170	504	45.4, 49.6,55.4
GPC-5 (100% FAH + 40% BAH)	0.7	0.4	12	1.5, 2, 2.5	85.16	125.74	388	230	1170	378	34.1, 35.4, 34.3
	0.7	0.4	14	1.5, 2, 2.5	66.2	141.1	388	230	1170	378	36.2, 36.5, 37
	0.7	0.4	16	1.5, 2, 2.5	55.4	150.3	388	230	1170	378	31, 31,37.2
GPC-6 (100% FAH + 50% BAH)	1	0.4	12	1.5, 2, 2.5	85.16	125.74	388	315	1170	315	26.2, 28.1, 29.1
	1	0.4	14	1.5, 2, 2.5	66.2	141.1	388	315	1170	315	25.5, 28, 25.1
	1	0.4	16	1.5, 2, 2.5	55.4	150.3	388	315	1170	315	25.5,29.6, 31.2

(FAH: Fly ash, BAH: Bottom Ash, CA: Coarse aggregates, FA: Fine aggregates, SS: sodium silicate, SH: sodium hydroxide, SS/SH: Sodium Silicate and Sodium hydroxide ratio, L/S: Precursor powder and Liquid ratio, CT: Curing time, S: Superplasticizer).

**Table 5:** Multilayer feedforward neural network models for the prediction of Compressive Strength of geopolymer concrete.

Designation	Algorithm	Number of Hidden Layers	Neurons in the Hidden Layer
ANN-I	Bayesian Regularization (BR)	1	5
ANN-II	BR	1	10
ANN-III	Levenberg-Marquardt (LM)	1	5
ANN-IV	LM	1	10
ANN-V	Scaled Conjugate Gradient (SCG)	1	5
ANN-VI	SCG	1	10
ANN-VII	BR	2	h_1_ = 10, h_2_ = 5
ANN-VIII	BR	2	h_1_ = 10, h_2_ = 10
ANN-IX	LM	2	h_1_ = 10, h_2_ = 5
ANN-X	LM	2	h_1_ = 10, h_2_ = 10
ANN-XI	BR	3	h_1_ = 10, h_2_ = 10, h_3_ = 5
ANN-XII	BR	3	h_1_ = 10, h_2_ = 10, h_3_ = 10
ANN-XIII	LM	3	h_1_ = 10, h_2_ = 10, h_3_ = 5
ANN-XIV	LM	3	h_1_ = 10, h_2_ = 10, h_3_ = 10

**Table 6:** Predicted GPC compressive strength for a new set of input parameters.

CT	S	L/S	SH	SS/SH	SH Concentration	SS Concentration	FAH	BA	CA	FA	Predicted CS
28	0.5	0.4	16	3	51.73	155.2	388	124	1170	504	61.4347889

## Data Availability

Not available.
